# Supplementary material for: Estimating the Effect of Motivational Interventions in Patients with Eating Disorders: A Systematic Review and Meta-Analysis
Source: J Pers Med. 2022 Apr 4;12(4):577. doi: 10.3390/jpm12040577 (PMC9028385; doi:10.3390/jpm12040577)
Supplement: Supplementary file 1 [file jpm-12-00577-s001.zip › jpm-1628160-Supplementary.pdf]

Supplementary Material

Table S1: Inclusion & Exclusion Criteria

	Inclusion criteria	Exclusion criteria
**Publication**	- Articles published in a peer-reviewed journal<br>- Grey literature	- Only abstract available<br>- Reviews<br>- Editorials<br>- Letters<br>- Meta-analyses<br>- Commentaries
**Intervention**	Motivational interventions	
**Exposure**	Diagnosed with an eating disorder in accordance with DSM or ICD-10 criteria e.g.:<br>● Anorexia nervosa (AN)<br>● Bulimia nervosa (BN)<br>● Binge eating disorder (BED)<br>● Other specified feeding or eating disorders (OSFED)<br>● Eating disorder not otherwise specified (EDNOS)<br>● Unspecified feeding or eating disorder (UFED)	Not diagnosed with an eating disorder in accordance with DSM or ICD-10.
**Comparison**	Control intervention or non-intervention	No control group
**Language**	Full text in English	
**Participants**	No restrictions on participant characteristics	
**Outcomes**	Primary outcome:<br>1. Change in level of motivation<br>Secondary outcome:<br>2. Change in ED symptomatology (BMI included)	
**Study design**	Randomized controlled trials	

Table S2: Search Terms

Database:	Search terms:	Results:
**MEDLINE (PubMed)**	(”Anorexia nervosa” OR "Anorexia Nervosa"[Mesh] OR "Bulimia Nervosa"[Mesh] OR Bulimia OR "Bulimia"[Mesh] OR Binge-Eating Disorder* OR "Binge-Eating Disorder"[Mesh] OR Feeding and Eating Disorder* OR "Feeding and Eating Disorders"[Mesh] OR EDNOS OR “Eating Disorder Not Otherwise Specified” OR OSFED OR “Other Specified Feeding and Eating Disorders” OR UFED OR “Unspecified Food and Eating Disorder”)<br>AND<br>("Motivational interviewing"[MESH] OR Motivational intervention* OR Motivational interview* OR Motivational enhancement therap* OR Motivational counseling OR Motivational counselling OR Motivational assessment* OR Behaviour change counselling OR Behavior change counseling OR Motivational level*)	975
**PsycInfo (EBSCO)**	#1: ((((DE "Eating Disorders") OR (DE "Anorexia Nervosa")) OR (DE "Binge Eating Disorder")) OR (DE "Bulimia")) OR (DE "Feeding Disorders")<br>#2: "Eating disorder" OR eating disorder* OR Anorexia OR "anorexia nervosa” OR "Binge-Eating Disorder" OR Bulimia OR “Bulimia Nervosa” OR "Feeding and Eating Disorders" OR Feeding and Eating Disorder* OR EDNOS OR “Eating Disorder Not Otherwise Specified” OR OSFED OR "Other Specified Feeding or Eating Disorder" OR UFED OR “Unspecified Feeding or Eating Disorders”<br>#3: (((DE "Readiness to Change") OR (DE "Motivational Interviewing")) OR (DE "Motivation Measures")) OR (DE "Motivation Training")<br>#4: Motivational interviewing OR mi OR motivational interview* OR Motivational intervention* OR motivation measure* OR motivational level* OR Motivational counseling OR Motivational counselling OR motivational enhancement therap* OR Motivational assessment* OR Behaviour change counseling OR Behaviour change counselling<br>#5: (#1 OR #2)<br>#6: (#3 OR #4)<br>#7: #5 AND #6	1.135
**Cochrane Library**	#1 "Eating disorder" OR eating disorder* OR anorexia nervosa OR bulimia nervosa OR bulimia OR Binge-Eating Disorder OR "Feeding and Eating Disorders" OR Feeding and Eating Disorder* OR EDNOS OR “Eating Disorder Not Otherwise Specified” OR OSFED OR "Other Specified Feeding or Eating Disorder" OR UFED OR “Unspecified Feeding or Eating Disorders”<br>#2 MeSH descriptor: [Anorexia Nervosa] explode all trees<br>#3 MeSH descriptor: [Bulimia Nervosa] explode all trees<br>#4 MeSH descriptor: [Binge-Eating Disorder] explode all trees<br>#5 MeSH descriptor: [Bulimia] explode all trees<br>#6 MeSH descriptor: [Anorexia] explode all trees<br>#7 MeSH descriptor: [Feeding and Eating Disorders] explode all trees	334

	#8 Motivational interviewing OR Motivational intervention* OR Motivational interview* OR Motivational enhancement therap* OR Motivational counseling OR Motivational counselling OR Motivational assessment* OR Behaviour change counselling OR Behavior change counseling OR Motivational level*<br>#9 MeSH descriptor: [Motivational Interviewing] explode all trees<br>#10 (#1 OR #2 OR #3 OR #4 OR #5 OR #6 OR #7) AND (#8 OR #9)	
**Embase (Ovid)**	#1 Eating disorder.mp. or exp eating disorder/<br>#2 eating disorder*.mp.<br>#3 anorexia nervosa.mp. or exp anorexia nervosa/<br>#4 bulimia nervosa.mp. or exp bulimia/<br>#5 (Feeding and Eating Disorder*).mp. [mp=title, abstract, heading word, drug trade name, original title, device manufacturer, drug manufacturer, device trade name, keyword, floating subheading word, candidate term word]<br>#6 EDNOS.mp.<br>#7 (Eating Disorder* not Otherwise Specified).mp. [mp=title, abstract, heading word, drug trade name, original title, device manufacturer, drug manufacturer, device trade name, keyword, floating subheading word, candidate term word]<br>#8 (Eating Disorder* not Otherwise Specified).mp. [mp=title, abstract, heading word, drug trade name, original title, device manufacturer, drug manufacturer, device trade name, keyword, floating subheading word, candidate term word]<br>#9 OSFED.mp.<br>#10 (Other Specified Feeding or Eating Disorder*).mp. [mp=title, abstract, heading word, drug trade name, original title, device manufacturer, drug manufacturer, device trade name, keyword, floating subheading word, candidate term word]<br>#11 UFED.mp.<br>#12 (Unspecified Feeding or Eating Disorder*).mp. [mp=title, abstract, heading word, drug trade name, original title, device manufacturer, drug manufacturer, device trade name, keyword, floating subheading word, candidate term word]<br>#13 1 or 2 or 3 or 4 or 5 or 6 or 7 or 8 or 9 or 10 or 11 or 12<br>#14 Motivational interviewing.mp. or exp motivational interviewing/<br>#15 Motivational intervention*.mp.<br>#16 Motivational interview*.mp.<br>#17 Motivational enhancement therap*.mp.<br>#18 Motivational counseling.mp.<br>#19 Motivational counselling.mp.<br>#20 Motivational assessment*.mp.<br>#21 Behaviour change counselling.mp.<br>#22 Behavior change counseling.mp.<br>#23 Motivational level*.mp.<br>#24 14 or 15 or 16 or 17 or 18 or 19 or 20 or 21 or 22 or 23<br>#25 13 and 24	194

Figures S1-S6: Sensitivity Analysis

	MI/MET			Control				Std. Mean Difference
Study or Subgroup	Mean	SD	Total	Mean	SD	Total	Weight	IV, Random, 95% CI
Cardi, 2019	−1.02	1.5	99	−1.03	1.58	88	24.5%	0.01 [−0.28, 0.29]
Dunn, 2006	−0.25	1.33	45	−0.04	1.47	45	14.6%	−0.15 [−0.56, 0.27]
MacDonald, 2017	−1.59	1.32	21	−2.23	1.2	23	7.8%	0.50 [−0.10, 1.10]
Schmidt, 2012	−1.21	1.97	34	−0.87	1.99	37	12.0%	−0.17 [−0.64, 0.30]
Schmidt, 2015	−0.32	1.74	72	−0.82	1.61	70	20.3%	0.30 [−0.03, 0.63]
Vella-Zarb, 2014	−0.76	1.52	24	−0.89	1.26	21	8.2%	0.09 [−0.50, 0.68]
Wade, 2009	−1.25	1.34	22	−0.82	1.3	25	8.4%	−0.32 [−0.90, 0.26]
Ziser, 2021	−2.1	1.69	11	−1.5	1.31	11	4.2%	−0.38 [−1.23, 0.46]
**Total (95% CI)**			**328**			**320**	**100.0%**	**0.02 [−0.16, 0.20]**
Heterogeneity: $Tau^2 = 0.01$; $Chi^2 = 8.65$, df = 7 (P = 0.28); $I^2 = 19\%$								
Test for overall effect: Z = 0.25 (P = 0.80)								

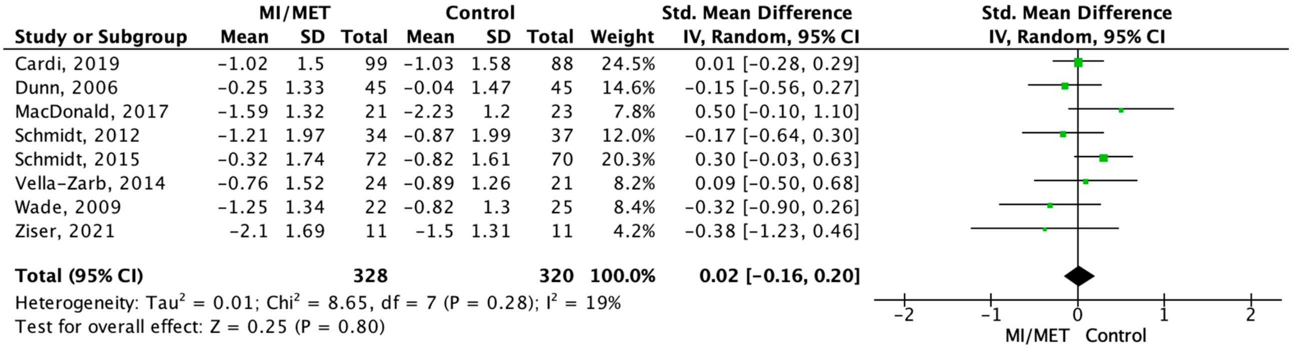

Figure S1: Forest plot showing the pooled effect of motivational enhancement therapy/motivational interviewing (MET/MI) on eating disorder psychopathology (EDP) using the standardized mean difference (SMD). Mean = mean change in EDP scores and the correlation coefficient (r) = 0.3.

	MI/MET			Control				Std. Mean Difference
Study or Subgroup	Mean	SD	Total	Mean	SD	Total	Weight	IV, Random, 95% CI
Cardi, 2019	−1.02	1.28	99	−1.03	1.35	88	20.9%	0.01 [−0.28, 0.29]
Dunn, 2006	−0.25	1.13	45	−0.04	1.25	45	14.7%	−0.17 [−0.59, 0.24]
MacDonald, 2017	−1.59	1.12	21	−2.23	1.03	23	8.9%	0.59 [−0.02, 1.19]
Schmidt, 2012	−1.21	1.68	34	−0.87	1.72	37	12.8%	−0.20 [−0.66, 0.27]
Schmidt, 2015	−0.32	1.48	72	−0.82	1.37	70	18.5%	0.35 [0.02, 0.68]
Vella-Zarb, 2014	−0.76	1.29	24	−0.89	1.07	21	9.4%	0.11 [−0.48, 0.69]
Wade, 2009	−1.25	1.26	22	−0.82	1.24	25	9.6%	−0.34 [−0.92, 0.24]
Ziser, 2021	−2.1	1.48	11	−1.5	1.11	11	5.2%	−0.44 [−1.29, 0.41]
**Total (95% CI)**			**328**			**320**	**100.0%**	**0.02 [−0.19, 0.23]**
Heterogeneity: $Tau^2 = 0.03$; $Chi^2 = 11.46$, df = 7 (P = 0.12); $I^2 = 39\%$								
Test for overall effect: Z = 0.20 (P = 0.84)								

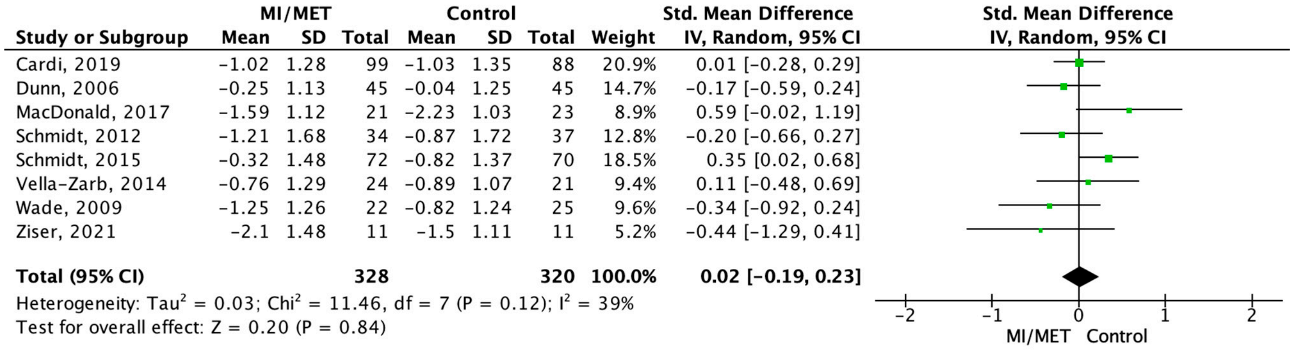

Figure S2: Forest plot showing the pooled effect of MET/MI on EDP using SMD with r = 0.5.

	MI/MET			Control				Std. Mean Difference
Study or Subgroup	Mean	SD	Total	Mean	SD	Total	Weight	IV, Random, 95% CI
Cardi, 2019	−1.02	0.84	99	−1.03	0.9	88	17.1%	0.01 [−0.28, 0.30]
Dunn, 2006	−0.25	0.71	45	−0.04	0.79	45	14.3%	−0.28 [−0.69, 0.14]
MacDonald, 2017	−1.59	0.72	21	−2.23	0.72	23	10.3%	0.87 [0.25, 1.49]
Schmidt, 2012	−1.21	1.09	34	−0.87	1.2	37	13.2%	−0.29 [−0.76, 0.18]
Schmidt, 2015	−0.32	1.31	72	−0.82	1.2	70	16.1%	0.40 [0.06, 0.73]
Vella-Zarb, 2014	−0.76	0.82	24	−0.89	0.72	21	10.9%	0.16 [−0.42, 0.75]
Wade, 2009	−1.25	1.15	22	−0.82	1.16	25	11.1%	−0.37 [−0.94, 0.21]
Ziser, 2021	−2.1	1.08	11	−1.5	0.72	11	7.1%	−0.63 [−1.49, 0.23]
**Total (95% CI)**			**328**			**320**	**100.0%**	**0.01 [−0.27, 0.29]**
Heterogeneity: $Tau^2 = 0.10$; $Chi^2 = 19.90$, df = 7 (P = 0.006); $I^2 = 65\%$								
Test for overall effect: Z = 0.07 (P = 0.94)								

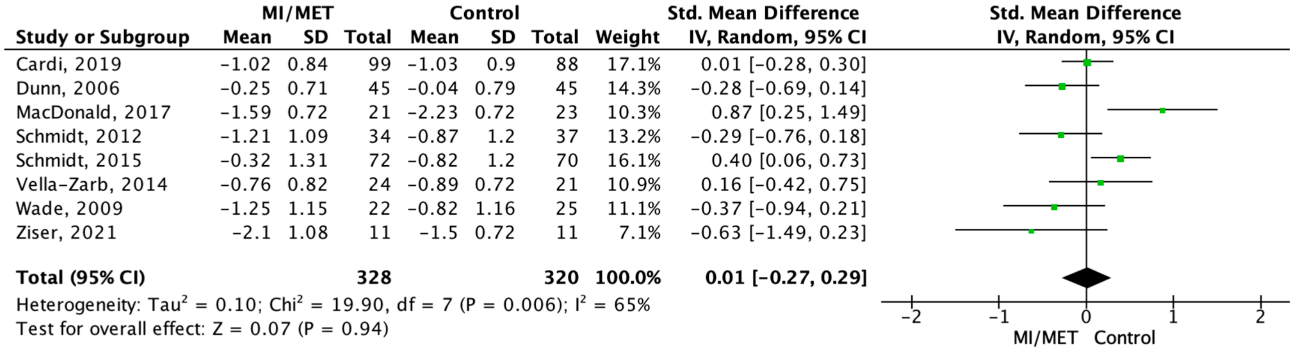

Figure S3: Forest plot showing the pooled effect of MET/MI on EDP using SMD with r = 0.8.

	MI/MET			Control				Mean Difference
Study or Subgroup	Mean	SD	Total	Mean	SD	Total	Weight	IV, Random, 95% CI
Cardi, 2019	1.1	2.58	99	1.23	2.19	88	32.2%	−0.13 [−0.81, 0.55]
MacDonald, 2017	0.4	6.86	21	0.2	6.98	23	0.9%	0.20 [−3.89, 4.29]
Schmidt, 2012	0.97	2.11	34	0.84	2.19	37	15.1%	0.13 [−0.87, 1.13]
Schmidt, 2015	1.07	2.17	72	0.81	2.17	70	29.6%	0.26 [−0.45, 0.97]
Wade, 2009	0.4	1.76	22	0.64	1.91	25	13.7%	−0.24 [−1.29, 0.81]
Ziser, 2021	1	1.38	11	1.2	1.77	11	8.6%	−0.20 [−1.53, 1.13]
**Total (95% CI)**			**259**			**254**	**100.0%**	**0.01 [−0.38, 0.39]**
Heterogeneity: $Tau^2 = 0.00$; $Chi^2 = 1.01$, df = 5 (P = 0.96); $I^2 = 0\%$								
Test for overall effect: Z = 0.03 (P = 0.97)								

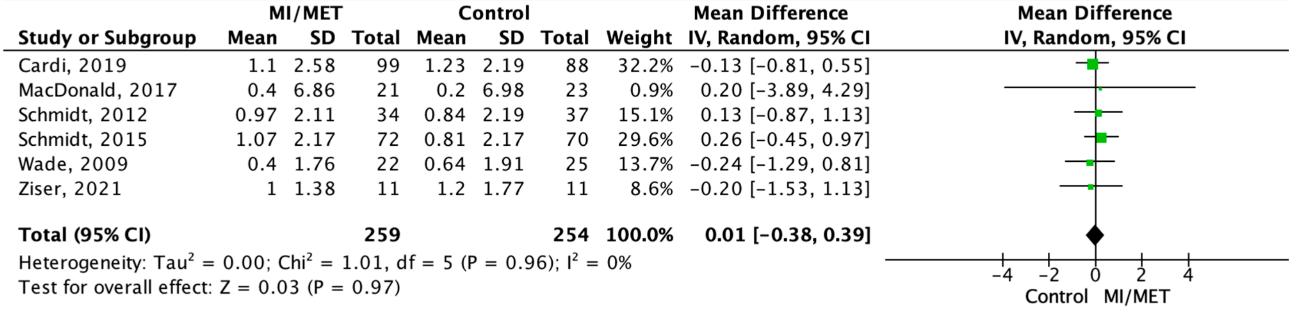

Figure S4: Forest plot showing the pooled effect of MET/MI on body mass index (BMI) using the mean difference (MD). Mean = mean change in BMI with r = 0.3.

	MI/MET			Control				Mean Difference
**Study or Subgroup**	**Mean**	**SD**	**Total**	**Mean**	**SD**	**Total**	**Weight**	**IV, Random, 95% CI**
Cardi, 2019	1.1	2.26	99	1.23	1.92	88	31.8%	−0.13 [−0.73, 0.47]
MacDonald, 2017	0.4	5.8	21	0.2	5.9	23	1.0%	0.20 [−3.26, 3.66]
Schmidt, 2012	0.97	1.84	34	0.84	1.91	37	15.0%	0.13 [−0.74, 1.00]
Schmidt, 2015	1.07	1.91	72	0.81	1.9	70	29.0%	0.26 [−0.37, 0.89]
Wade, 2009	0.4	1.51	22	0.64	1.61	25	14.3%	−0.24 [−1.13, 0.65]
Ziser, 2021	1	1.18	11	1.2	1.5	11	9.0%	−0.20 [−1.33, 0.93]
**Total (95% CI)**			**259**			**254**	**100.0%**	**0.00 [−0.33, 0.34]**

Heterogeneity: $Tau^2 = 0.00$; $Chi^2 = 1.34$, df = 5 (P = 0.93); $I^2 = 0\%$
Test for overall effect: Z = 0.02 (P = 0.98)

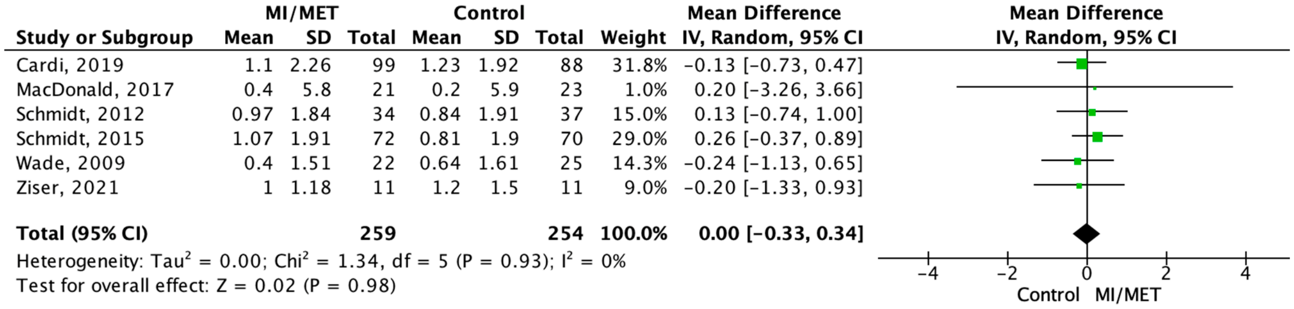

Figure S5: Forest plot showing the pooled effect of MET/MI on BMI using the MD with r = 0.5.

	MI/MET			Control				Mean Difference
**Study or Subgroup**	**Mean**	**SD**	**Total**	**Mean**	**SD**	**Total**	**Weight**	**IV, Random, 95% CI**
Cardi, 2019	1.1	1.69	99	1.23	1.43	88	29.2%	−0.13 [−0.58, 0.32]
MacDonald, 2017	0.4	3.67	21	0.2	3.74	23	1.2%	0.20 [−1.99, 2.39]
Schmidt, 2012	0.97	1.32	34	0.84	1.39	37	14.7%	0.13 [−0.50, 0.76]
Schmidt, 2015	1.07	1.44	72	0.81	1.38	70	27.1%	0.26 [−0.20, 0.72]
Wade, 2009	0.4	1.04	22	0.64	1.02	25	16.7%	−0.24 [−0.83, 0.35]
Ziser, 2021	1	0.78	11	1.2	0.95	11	11.1%	−0.20 [−0.93, 0.53]
**Total (95% CI)**			**259**			**254**	**100.0%**	**−0.01 [−0.25, 0.23]**

Heterogeneity: $Tau^2 = 0.00$; $Chi^2 = 2.65$, df = 5 (P = 0.75); $I^2 = 0\%$
Test for overall effect: Z = 0.07 (P = 0.95)

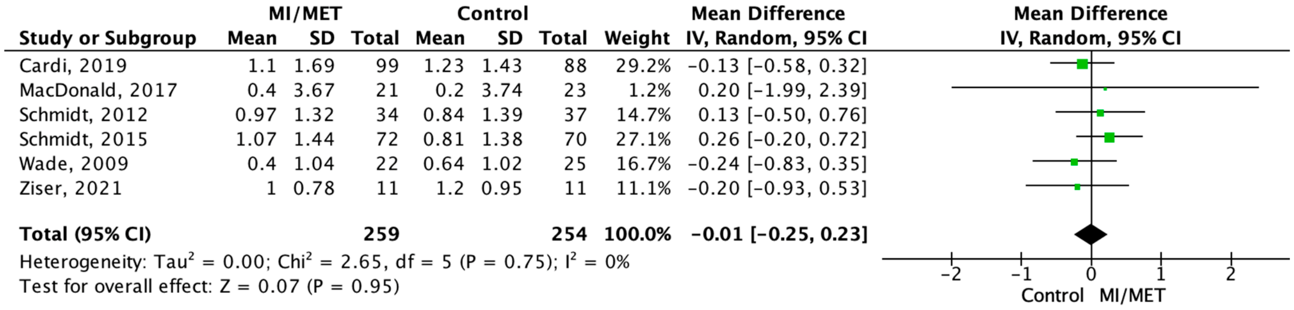

Figure S6: Forest plot showing the pooled effect of MET/MI on BMI using MD with r = 0.8.

Figures S7-S9: Meta-Regression Analysis

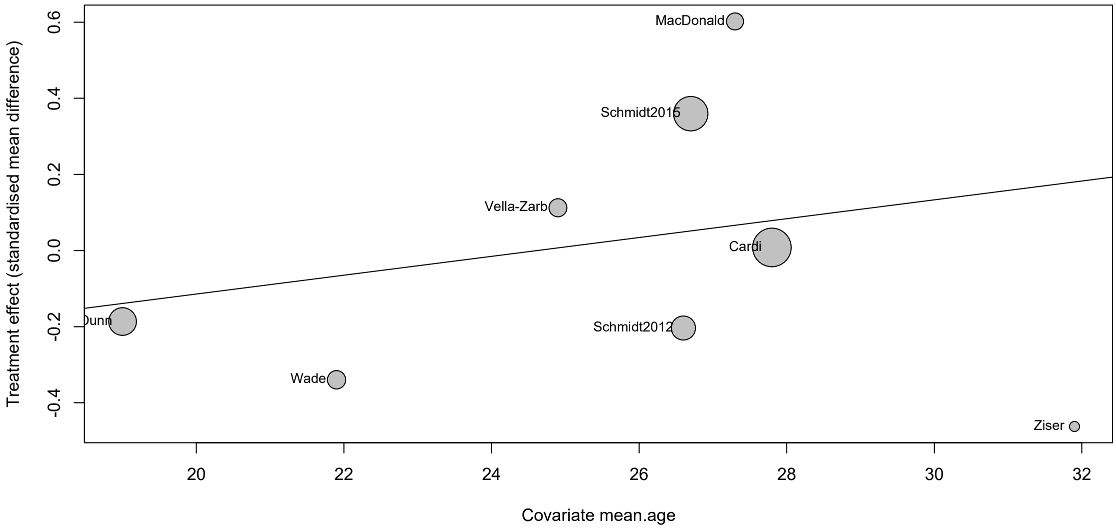

Figure S7: Meta-regression using mean age as a moderator.

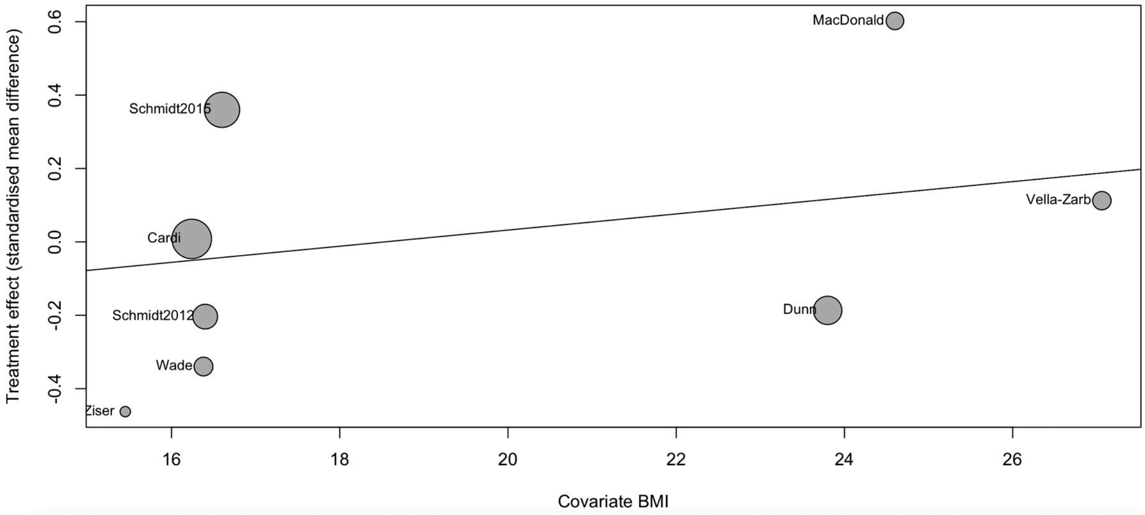

Figure S8: Meta-regression using BMI as a moderator.

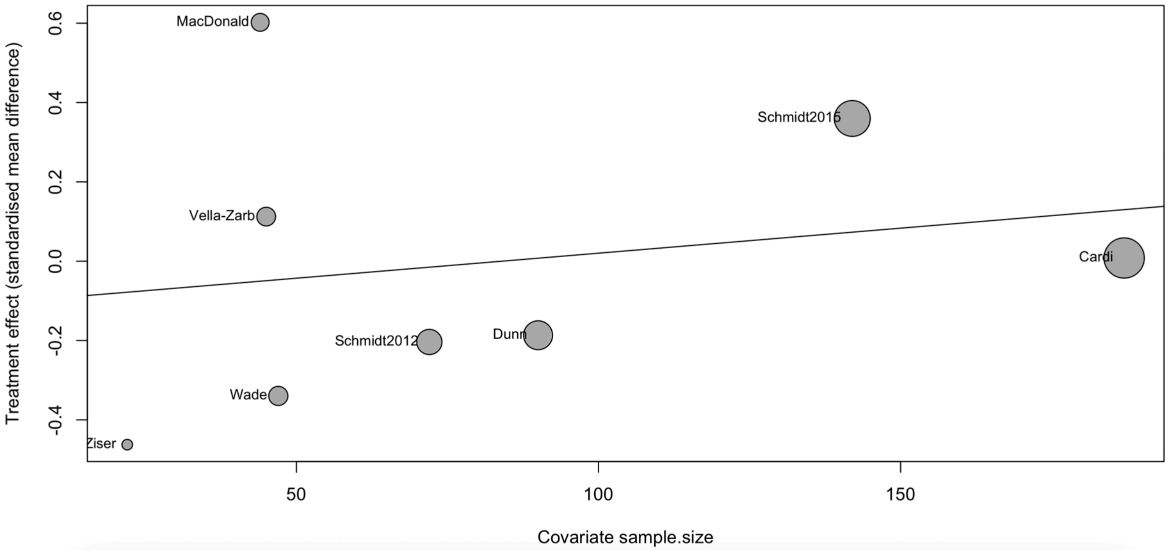

Figure S9: Meta-regression showing sample size as a moderator.

Tables S3-S5: Meta-Regression Analysis

Table S3: Meta-regression analysis using mean age as a moderator.

	Value	SE	t-value	df	p-value	Lower CI	Upper CI
**Intercept**	-0.6089	0.8938	-0.6813	6	0.5211	-2.7959	1.5781
**Slope**	0.0247	0.0348	0.7112	6	0.5036	-0.0604	0.1098
**R^2**	0.00 %	-	-	-	-	-	-
**H^2**	1.69 %	-	-	-	-	-	-
**I^2**	40.80 %	-	-	-	-	-	-
**Tau^2**	0.0379	0.0550	-	-	-	-	-
**Tau**	0.1947	-	-	-	-	-	-

Table S4: Meta-regression analysis using mean BMI as a moderator.

	Value	SE	t-value	df	p-value	Lower CI	Upper CI
**Intercept**	-0.4083	0.5603	-0.7287	6	0.4937	-1.7793	0.9627
**Slope**	0.0220	0.0284	0.7766	6	0.4669	-0.0474	0.0914
**R**	0.00 %	-	-	-	-	-	-
**H^2**	2.06	-	-	-	-	-	-
**I^2**	51.35 %	-	-	-	-	-	-
**Tau^2**	0.0567	0.0664	-	-	-	-	-
**Tau**	0.2382	-	-	-	-	-	-

Table S5: Meta-regression analysis using sample size as a moderator.

	Value	SE	t-value	df	p-value	Lower CI	Upper CI
**Intercept**	-0.1060	0.2450	-0.4327	6	0.6804	-0.7055	0.4935
**Slope**	0.0013	0.0022	0.5819	6	0.5818	-0.0040	0.0066
**R**	0.00 %	-	-	-	-	-	-
**H^2**	1.89	-	-	-	-	-	-
**I^2**	47.07 %	-	-	-	-	-	-
**Tau^2**	0.0518	0.0658	-	-	-	-	-
**Tau**	0.2277	-	-	-	-	-	-
